# 12 TIPS for Implementing Peer Instruction in Medical Education

**DOI:** 10.15694/mep.2020.000237.1

**Published:** 2020-10-23

**Authors:** Dean Parmelee, Mary Jo Trout, Irina Overman, Michael Matott

**Affiliations:** 1 Wright State University Boonshoft School of Medicine

**Keywords:** Active learning, peer-to-peer teaching, collaborative learning, immediate feedback for learner, 'just in time' teaching, continuous assessment of learning, formative assessment, retrieval-based practice, multiple choice questions

## Abstract

This article was migrated. The article was marked as recommended.

Peer Instruction (PI) is a vibrant instructional strategy, used successfully for over two decades in undergraduate physics and mathematics courses. It has had limited use and few publications in medical education. This 12 TIPS provides a focused review on the evidence supporting its use in higher education and rationale for its wider adoption in medical education. The authors detail important steps for its implementation with large classes. Based on several years of experience with PI in a US allopathic medical school, they feel that PI attends to core principles from the science of learning and provides students and faculty with immediate feedback on learning. It is also adaptable to on-line synchronous administration.

## Introduction

With the publication of ‘Educating physicians for the future: Carnegie’s calls for reform’ ( Irby, 2011), undergraduate medical education initiated a number of pedagogical transformations to enhance the life-long learning skills of future physicians. Active learning strategies were adopted and adapted from college-level instruction to promote learner-centered education and reduce lecture hours for medical students. One strategy that has not been adopted widely in undergraduate medical education is Peer Instruction (PI), which is odd since its learning outcomes in physics and mathematics education are robust ( Crouch and Mazur, 2001; Freeman *et al.*, 2014).

Developed for undergraduate physics classes at Harvard by Professor Eric Mazur in the 1990’s ( Mazur, 1997), PI is likely the first strategy to incorporate what was then the ‘new’ technology of the Audience Response System (ARS), or clickers, and the associated performance data from these devices. Professor Mazur wisely adapted his strategy over time as he learned more about classroom dynamics and the power of peer-to-peer learning when structured through his evolving strategy. PI expanded widely within the science, technology, engineering, and math (STEM) fields and to a more modest degree in the biological sciences. Instructors of college-level molecular biology have demonstrated its impact on the quality of peer-peer discourse that leads to improved reasoning and how instructor facilitation enhances peer-peer learning ( Knight, Wise and Southard, 2013; Knight, Wise and Sieke, 2016; Knight and Brame, 2018). Vickrey *et al* (2015) provide a comprehensive review of the PI model for STEM fields and its effectiveness, instructor and learner perspectives, and articulate the necessary and critical elements for its implementation supported by evidence ( Vickrey *et al*, 2015).

For the medical education literature, Rao and DiCarlo (2000) published the use of PI for the respiratory section of a medical school physiology course, with results suggesting that PI “enhances the quantity, depth, and detail of material covered...,” shows greatest improvement in knowledge is in synthesis and evaluation skills questions, and is an effective learning strategy for large classes ( Rao and DiCarlo, 2000, p 55). Giuliodori, Lujan and DeCarlo (2006) demonstrated that veterinary medicine students performed better on physiology qualitative problem-solving questions when PI was the instructional strategy ( Giuliodori, Lujan and DeCarlo, 2006). Trout and colleagues, using a modified approach to PI, showed improved exam scores in a medical school pharmacology course ( Trout, Borges and Koles, 2014). Versteeg *et al* (2018), using first year medical students, demonstrated PI improved comprehension and transfer of physiological concepts, and that more students select correct answers after discussion regardless of their initial answers ( Versteeg *et al*, 2018). Passeri and colleagues used PI for a review session in three medical school pre-clinical subjects comparing it to a control group that had individual feedback from the instructor if requested; the PI group retained significantly more basic science knowledge on an exam given to both groups six months later ( Passeri, Mazur and Passeri, 2019).

The following 12 Tips guides instructors in the development, implementation, and evaluation of PI in medical education settings. They are derived from several years of experience at Wright State University Boonshoft School of Medicine in both the pre-clinical (75% of classroom time) and clinical phases. Team-Based Learning (TBL) and modified problem-based learning make up the remaining pre-clinical weekly classroom time, set at a maximum of 12 hours; there are no lectures across the four years.

## TIP 1: Determine the out-of-class resources students need to study to engage with your questions.

**Figure 1.**
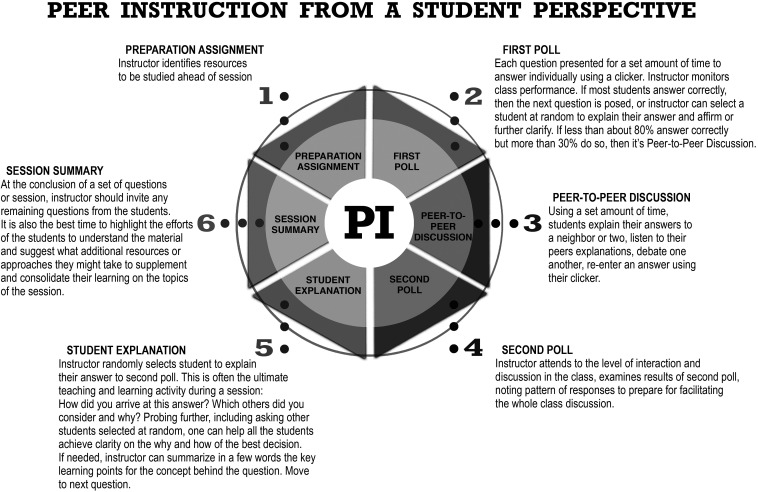
A schematic of Peer Instruction from the student perspective

This is the ***Preparation Assignment*** ( Figure 1, #1) ***.*** Unlike a summative assessment, you are not expecting the students to master the material before class. They will engage in the important learning processes of retrieval and self-explanation to answer the 1 ^st^ poll ( Figure 1, # 2); this prepares them for the next step of explaining their answer to a peer, listening to peers’ explanations, reconsidering options through discourse, before entering the 2 ^nd^ poll. It is a ‘good sign’ if the learners experience and express confusion, uncertainty, lack of confidence with the assignment at the beginning of a session, for this guarantees high engagement in the session as they struggle to apply the material to the questions through discourse and argumentation. Over time, students will increase their efforts with the ***Preparation Assignment*** because they want to engage more fully in the discourse with peers.

## TIP 2: Focus learning objectives on the application of learning.

Follow instructional design principles to create learning objectives that focus the learner’s effort on “what will I be able to do from learning this.” Clarify, through examples, what that would be and how it is also an assessment. See Table 1.

**Table 1.** Make learning objectives active with measurable outcomes

Topic	Application of learning	Example
Autonomic Drugs	Given a series of laboratory or clinical case vignettes, be able to select the best agent and explain how/why it works at the molecular/cellular/systems level.	An experimental vignette of a patient given norepinephrine. Predict appropriate effects on SBP, DBP, MAP, and HR...

## TIP 3: Convert the classroom to a ‘laboratory’ with the audience-response system (ARS).

Every class session can become a ‘laboratory’ for the instructor to evaluate and improve the questions so that they generate deep and spirited discussion. The clicker, a tool of the ARS, requires each student to ‘commit’ to an answer, then defend that answer with a neighboring peer or two or even three. Sometimes, the student will hear a better explanation from a peer, or a peer will question his/her rationale and thereby think differently about their own answer. Then, the student enters the second poll with the same or a different answer. The instructor watches the class interacting between the first and second poll, taking note of the noise volume and the amount of psychomotor activity as students explain their thinking to one another - and, at the same time, watches the polling data change on the ARS screen, almost invariably towards the correct answer.

It is important to select an ARS that makes it easy to import questions and still allow last minute editing. Polling results, as they evolve, should be clearly visible to the instructor and any grade recording from the polling should be easily transferable to the course grade book. We easily found an ARS that worked well in the on-line, synchronous learning environment and coordinated it with an on-line platform that creates small groupings of students.

## TIP 4: Write primarily multiple-choice questions (MCQs) that force thinking.

This is undoubtedly the most difficult task in creating a PI session and the most critical. We suggest, as part of the instructional design process, crafting a detailed outline of the ***Preparation Assignment***; its elements build a scaffold for the learning of the topic. Focus your questions on achieving these learning outcomes. A question that demands interpretation, calculation, deep thinking (not simple recall) will generate discussion around the set of plausible options; posing two options can be sufficient to stir-up considerable debate.

**Figure 2.**
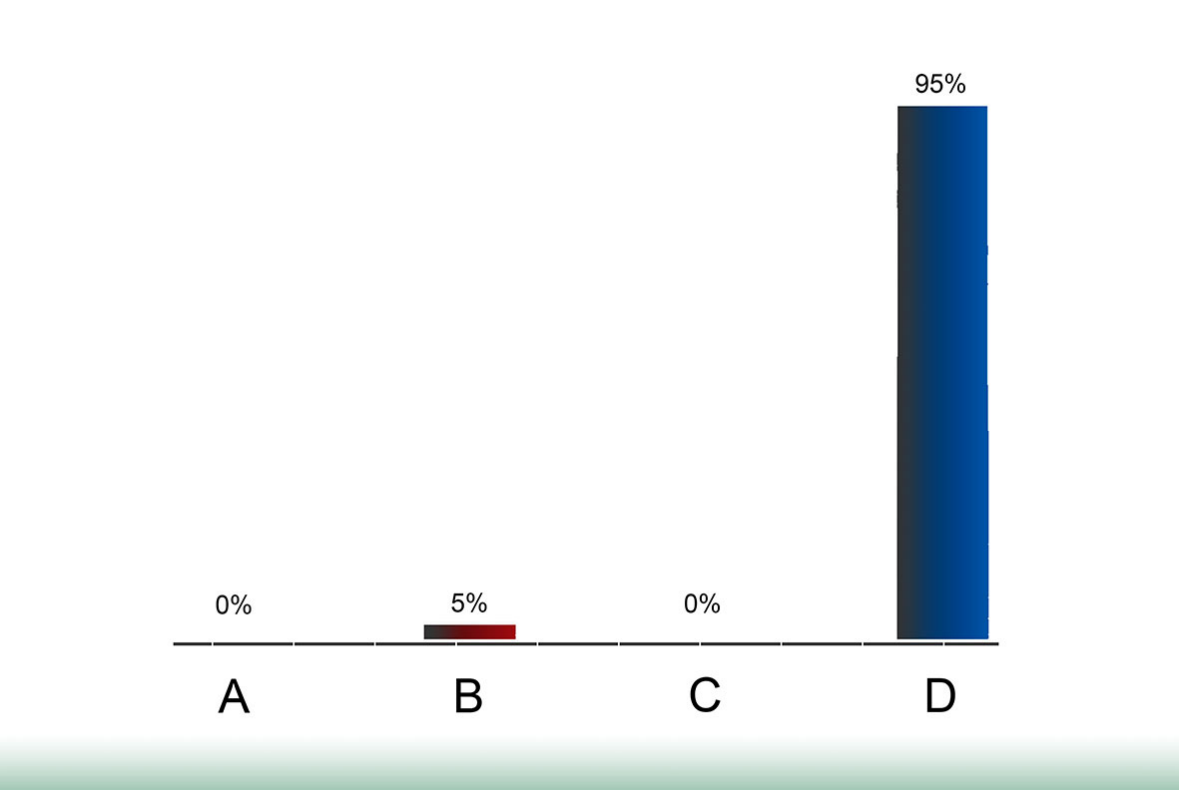
First poll showing no need for discussion

**Figure 3.**
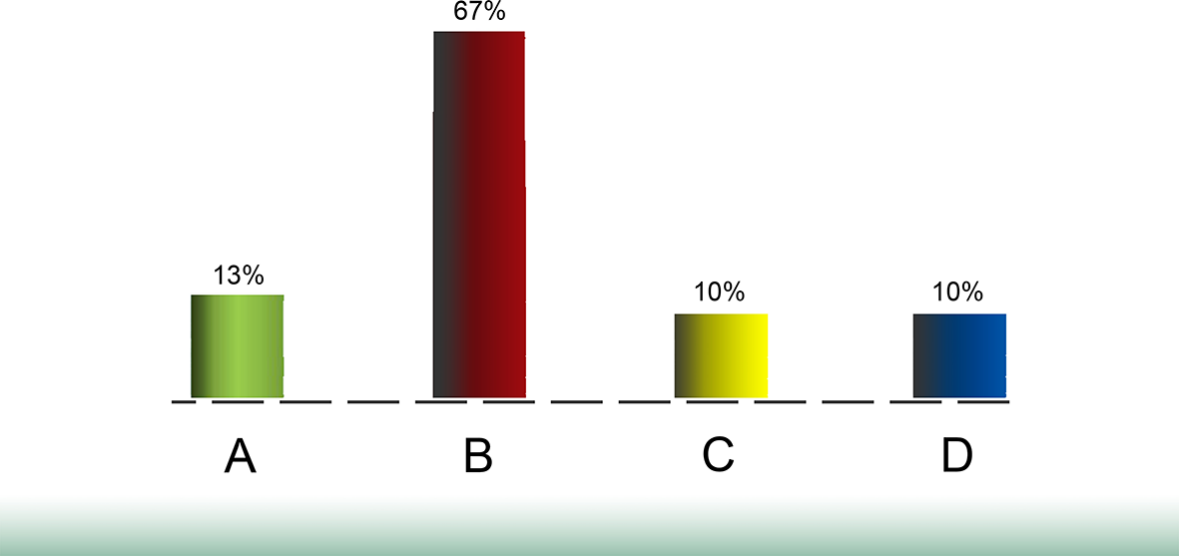
First poll showing no need for Peer-to-Peer discussion

For a particularly difficult topic, create more than one question on a concept from a different perspective or different data set; this reinforces the students’ retrieval-based application of learning. Sequence the questions to help build their knowledge of these difficult topics. Use simple recall questions very sparingly because these do not typically go to discussion and 2 ^nd^ poll. Since discussion and argumentation is where the greatest learning takes place, it is important to have >90% of questions go to 2 ^nd^ poll. If time permits, before moving on after a challenging question, ask “what was the learning objective of this question?” See Figures 2 & 3.

## TIP 5: Determine a grading schema and time management routine.

Our students assisted in the initial design of how much each poll would ‘count’ as part of a course grade (Yelton *et al*, 2014). It works well if the 1 ^st^ poll carries a very modest point value and if at least 80% of the class select the correct answer. According to the students, knowing that every question can have point value incentivizes them to prepare for and attend class. When the 1 ^st^ poll results indicate that less than 80% have chosen the correct answer, it is the opportunity for ***Peer-to-Peer Discussion*** ( Figure 1, #3), leading to a 2 ^nd^ poll. After this, each student must individually answer the question. Since peer-to-peer collaboration and engaged discourse is a key goal, we only grade the individual answer to the second poll when used.

On average, PI scores compose a small percentage of a student’s course grade. 2 ^nd^ poll results generally show 80-100% correct, indicating wide class understanding of the question. Because all of our students must take a high-stakes exam for licensure, we allow 1.2 minutes per question for the 1 ^st^ poll and 2 minutes for the ‘discussion’ before the 2 ^nd^ poll, though on occasion we extend this time when we feel the question needs more discourse opportunity. We structure our PI sessions into 3-hour blocks, with 10-minute breaks every hour; usually 8-10 questions per hour. Only one session in a day. If the learning objectives for each PI session are derived from the course objectives and applied to each question, then students will be prepared for the course’s summative examination.

## TIP 6: Orient the class by having them participate with a few sample PI questions.

The best introduction is to do a mini-PI session, with 2-3 questions, derived from a simple assignment they can read in class; display Figure 1 or a similar image as a guide. Show the class the results of the 1 ^st^ poll and how you make the decision to move onto ***Peer-to-Peer Discussion*** and 2 ^nd^ poll. Demonstrate a process whereby you select **at random** a student to explain their answer choice and why other choices may be incorrect. Reassure the class that responses such as “I have no idea” or “I went with what John (neighbor) picked” are acceptable---honesty is valued; move on to the next person or ask what did they think made it difficult to answer. Emphasize how important it is to fully engage with peers when a question goes to ***Peer-to-Peer Discussion*** by explaining one’s answer, listening to others explain their rationale for the answer, debate, ask each other more questions, work with each other to ‘figure it out.’ Encourage them to ask questions of the instructor when he or she says “Are there more questions about this one.” Avoid launching into any detailed explanation or presentation other than to clarify misconception.

## TIP 7: Request other faculty and students, who have taken the course, to evaluate and critique new questions.

They can help you check spelling, grammar, and evaluate whether or not a question will generate good discussion. Such review will also prepare you for how to facilitate the discussion of questions that address difficult or confusing concepts. The instructor increases the depth of discussions between students by cueing them to focus on their ‘reasoning’ for an answer rather than the answer ( Knight, Wise and Southard, 2013).

## TIP 8: During the Peer-to-Peer Discussion, listen in to conversations, observe the interactions.

Attentively glean all you can from this period when the students are engaged in discourse about a question, defending their explanations to one another, working hard to get to the best answer.During this phase and when a student is explaining an answer to the class is when you learn how your students are thinking about the course content and concepts. As they enter their second poll selections, you monitor the shifts in their thinking about the questions - usually about 90% of students will enter the correct answer following this peer-to-peer teaching and learning.

## TIP 9: During Student Explanation, probe explanations – the WHY and HOW.

If a student stumbles, affirm that this is part of learning, give praise of the effort, and select another student to move the discussion along. If you hear two good opposing views, generate a debate in the class on the spot. You can even go to a 3 ^rd^ poll if the class becomes split and you feel both positions need further discussion in the smaller clusters. It is during these interchanges with the students, as you and they explore rationales that raise more questions, that your role as *teacher* is most evident. From time-to-time, highlight how a particular question links to the learning objectives.

## TIP 10: Keep any instructor explanations to a minimum in number and duration.

Most commonly, when you show a PowerPoint slide and present more than a 1-2 minute explanation, you will lose student attention. They will learn more if you probe the class for their explanations and rationales before giving your clarifications. If you have prepared ‘teaching’ slides in case they are needed to emphasize key concepts in graphic representation, distribute these after the class.

## TIP 11: Complete sessions a few minutes early.

Ask two or three students to identify the single concept that they feel they learned from the session - that they didn’t know beforehand. This helps students feel connected to fellow students and gives them insight into how their peers are grappling with the material. Conclude with a statement on what you learned from the session about how they are working with the material. If you feel they need to dig deeper in the reading, let them know that you expect them to struggle with it and that’s the best way to learn. If they worked hard during the session, posing good arguments with one another during ‘discussion’ and had tough questions for you, compliment them.

## TIP 12: Evaluate a completed session as soon as possible.

Unlike a lecture presentation, when you have finished a PI session you have data on what and how your students have learned, or not. The data from first and second polls can be used to identify students who are struggling, informs you about the overall class knowledge level, and lets you know what questions worked well, at least statistically. The volume of talking, level of psychomotor activity, and quality of discourse between students during the ***Peer-to-Peer Discussion***, along with the depth of the questions they ask after the ***Student Explanation*** gives you rich additional information on each question.

## Conclusions

Peer Instruction is a remarkably interactive and powerful teaching and learning strategy for the classroom, and, from our recent experience during the COVID-12 pandemic, for the synchronous on-line platform. As with all ‘flipped classroom’ strategies, the quality of planning and preparation by the instructor is critical for its success. A user- and instructor- friendly audience response system is an enormous benefit for providing valuable attendance and performance data for the instructor and immediate feedback for the student. Student feedback on Peer Instruction’s social interactive dimension and content learning will make this strategy a more common one in medical education.

## Take Home Messages

• Peer Instruction is instructor guided and a learner-centered strategy.
• It provides learners with immediate feedback on their learning and instructors feedback on learner progress.
• Every session can become learning ‘laboratory’ for both instructor and learner. As instructor, pay attention to how learners are thinking and discussing.
• Peer-to-Peer discussions are enhanced with instructor guidance, such as “Consider the driving concept in this question, articulate your thoughts but listen to your peers.”
• Change learner seating assignments regularly to prevent social clustering; call on learners randomly to explain reasoning, not just give the answer

## Notes On Contributors

**Dean Parmelee**, MD, is Director of Educational Scholarship and Program Development, Professor of Medical Education, Psychiatry & Pediatrics at the Wright State University Boonshoft School of Medicine. He works with faculty and students to enhance the scholarship of teaching and learning, and consults with institutions of higher learning on curricula innovation. He was awarded the AOA Robert J. Glaser Distinguished Teacher Award in 2016 by the Alpha Omega Alpha Honorary Medical Society and the American Associate of Medical Colleges.

**Irina Overman**, MD is Assistant Professor of Internal Medicine, Geriatrics, and Medical Education, and serves as Director of Foundations of Clinical Practice in the WrightCurriculum at the Wright State University Boonshoft School of Medicine. She also directs two of the modules within the Foundations phase and champions the use Peer Instruction in medical education.

**Mary Jo Trout**, PharmD is Assistant Professor of Pharmacology & Toxicology, Geriatrics, and Medical Education, Director of the Therapeutics Curriculum at the Wright State University Boonshoft School of Medicine.

**Michael Matott**, PhD is Assistant Professor of Neuroscience, Cell Biology and Physiology at the Wright State University Boonshoft School of Medicine. He co-directs *Staying Alive*, the school’s biomedical science module on cardiovascular, respiratory, and renal systems.
