# Supplementary material for: 12 TIPS for Implementing Peer Instruction in Medical Education
Source: MedEdPublish (2016). 2020 Oct 23;9:237. [Version 1] doi: 10.15694/mep.2020.000237.1 (PMC10697530; doi:10.15694/mep.2020.000237.1)
Supplement: Peer review history

# Reviewer report: Reviewer response for version 1

**DOI:** 10.21956/mep.20175.r31191

**Published:** 2021-04-20

**Authors:** Barbara Jennings

**Affiliations:** 1 Norwich Medical School

This review has been migrated. The reviewer awarded 4 stars out of 5 I enjoyed reading this article and will recommend it to colleagues in faculty-development sessions about large group teaching to emphasise the importance of enhancing learning gain.There are three particularly strong aspects to the article in my opinion1. The discussion of the key experimental literature about using PI from Physics education and then its relevance to STEM subjects in general. 2. The tips on how to create psychologically safe classrooms – which is critical to the success of this educational approach3. And importantly, a thorough description of how to manage classes with PI based on several years of experience in both the pre-clinical and clinical teaching. I wonder if the low uptake of this robust method in medical education (as noted by the authors) is because teachers needed this type of step by step protocol?My only suggestions to improve the article would be to make figure 1 less busy with more succinct descriptors or headings – to improve clarity. And to take care with figure titles and legends – also I’m not sure the information in table 1 needed tabulation.

Reviewer Expertise:

NA

No decision status is available

# Reviewer report: Reviewer response for version 1

**DOI:** 10.21956/mep.20175.r31192

**Published:** 2021-01-18

**Authors:** Holly Bender

This review has been migrated. The reviewer awarded 5 stars out of 5 This well written, informative, 12 TIPS article introduces medical educators to a specific type of Peer Instruction (PI), first developed by Eric Mazur for his undergraduate physics and math courses. PI is an instructor-facilitated active-learning strategy that uses small groups, a “flipped-classroom approach,” and immediate feedback via an audience response system (clicker). The authors are widely recognized in the medical education community for implementing and disseminating other evidence-based, student-centered, active learning strategies such as Team-Based Learning and Problem Based Learning. Here, the authors make a compelling case for PI being an effective, engaging, evidence-based educational strategy and why PI should be adopted widely in medical education. The introduction cites relevant educational research from key manuscripts in multiple disciplines demonstrating improved student problem-solving skills, exam scores, and other positive learning outcomes associated with PI use. Their 12 tips offer a concise description of PI supported by a well-designed graphic illustrating the multi-step process. This combination makes it clear to educational practitioners the "nuts and bolts" of implementing PI in their classrooms. This article is especially relevant in the days of COVID as medical educators strive to keep students engaged online. Initially developed for large in-person classes, the authors describe how to give students immediate feedback for answering well-designed PI questions and facilitate engaging peer-to-peer discussions. The approach used in the manuscript makes it a must-read for busy educational practitioners who are looking for a practical and straightforward overview of PI, how to get up to speed to adopt the method quickly and how to leverage PI to evaluate student learning. The authors also reference further reading for those who would like to dig a little deeper into the literature.I have a minor suggestion. I believe Figure 3 is mislabeled and should instead say: Figure 3: First poll showing the need for Peer-to-Peer discussion

Reviewer Expertise:

NA

No decision status is available

# Reviewer report: Reviewer response for version 1

**DOI:** 10.21956/mep.20175.r31194

**Published:** 2020-12-24

**Authors:** Ken Masters

**Affiliations:** 1 Sultan Qaboos University

This review has been migrated. The reviewer awarded 3 stars out of 5 A useful and interesting paper on peer-instruction (PI) and how to implement it. The paper has several useful practical ideas on the topic.There are, though, two broad areas that really do need to be addressed:• Dealing with the contextual teaching culture. I see that the authors have based their experience largely in an environment in which no lectures occur. The transition to that state must have been an enormous undertaking; once done, however, introducing something like PI is surely easier than if one wished to introduce it to an environment where lectures (perhaps supplemented with tutorials, which might also be little more than didactic teaching in small groups) are the norm. When the entire pedagogical model and administration system relies on lectures, then the change is an uphill struggle: not only from the side of the institution, but also from the students who demand notes that can be learned and then regurgitated in the exams. Even when they do participate in PI, they are tempted to wait until the “the answer” can be given to them. So, what is the process to overcome this institutional inertia?• Related to the previous point, balancing the anticipated success with reasonable expectations. While the authors are enthusiastic about the process, the danger is that their enthusiasm gives the novice the impression that the process is relatively easy. In truth, education is littered with wrecks of good ideas and methods (IPE, PBL, TBL) that were enthusiastically yet poorly implemented because of over-confidence based on the very successful models and examples (Failures do not easily get published). While these have all been done properly and well, there are also many educators who have been burnt because they misunderstood the complexities and hard work required, and so they have reverted to the more comfortable didactic lecture. A simple example of a possible problem is knowing that MCQs promoting critical thinking should not be re-used from year to year, and need to be re-created each year. The reason is that students share the questions with the next year’s students, and then those students simply learn the question and answer without any form of understanding (e.g. “The question about the nurse and the patient with leukaemia, the answer is 15.”). In addition, saying that the initial student confusion is a “good sign” does not help the struggling lecturer trying to cope with the outrage of 150 angry students who feel that they have been short-changed, and complain on social media that the new teaching method means they have to do all the work and the lecturers get paid for saying “I don’t know.” So, to be fair to the readers, I really would like to see the authors identify the likely unanticipated problems, and how to prevent, or at least mitigate, them.I realise that a 12-tips paper is short, and one cannot go into these in much detail, but they do need to be identified, acknowledged, and then addressed to some extent. I look forward to Version 2 of the paper in which this is performed.

Reviewer Expertise:

NA

No decision status is available

# Reviewer report: Reviewer response for version 1

**DOI:** 10.21956/mep.20175.r31193

**Published:** 2020-10-29

**Authors:** Felix Silwimba

**Affiliations:** 1 University of Lusaka

This review has been migrated. The reviewer awarded 5 stars out of 5 the title is consistent with the contents of the script. the paper is original and relevant to medical education in this covid -19 error. since the students have to learn from home. it is very important that lectures facilitate this peer instructions and the suggested 12 points are valid everywhere .

Reviewer Expertise:

NA

No decision status is available

# Reviewer report: Reviewer response for version 1

**DOI:** 10.21956/mep.20175.r31190

**Published:** 2020-10-29

**Authors:** Sarmishtha Ghosh

**Affiliations:** 1 International Medical University

This review has been migrated. The reviewer awarded 4 stars out of 5 This is one very important "12-tips" paper giving structured guidelines to "PeerPeer Instruction" as an essential and important learner-centered yet facilitator guided strategy.Peer learning has been recognized as effective since the question of a threatened environment is minimized. Peer to peer conversation opens up a dialogue and leads to learning rather than a teacher dictating content to the students. However, some students have an objection to learning from peers that means someone at his own level. They often argue in favor of this viewpoint.So the term " Peer Instruction" needs some reviewing to make it more meaningful and contextual. It is basically Peer learning but guided by an Instructor or to be more specific a facilitator of learning.The tips are well explained and are mostly in lines of Flip Class, or even Team-Based Learning.The fact that is worth mentioning here is irrespective of the terms, these tips can be used with appropriate modifications to ensure learning in students at various levels of their training. The paper is useful for trainers and students.

Reviewer Expertise:

NA

No decision status is available
